# A Case Report With Dual Diagnosis of Candidiasis in a Patient With Uncontrolled Diabetes Mellitus

**DOI:** 10.7759/cureus.65908

**Published:** 2024-07-31

**Authors:** Komal V Dadgal, Swapnil Mohod

**Affiliations:** 1 Oral Medicine and Radiology, Sharad Pawar Dental College and Hospital, Datta Meghe Institue of Higher Education and Research, Wardha, IND

**Keywords:** infections in immunocompromised, multiple recurrent oral plaque-like lesions, oral plaque-like lesions, diabetes mellitus, immuno suppresion, oral candidiasis, uncontrolled hyperglycemia, oral thrush

## Abstract

Chronic hyperglycemia is a hallmark of diabetes mellitus (DM), one of the most common endocrine illnesses affecting millions of people globally. A key issue for diabetes patients is a compromised immune system, which impairs their capacity to fight off invading microbes and increases their susceptibility to infections. Compared to the healthy population, those with DM experience noticeably longer recovery from illnesses or injuries. Individuals suffering from DM are more susceptible to Candida albicans colonizing their oral and/or vaginal mucosa and urinary system. The present article presented a case of a 52-year-old female patient who reported recurrent multiple plaque-like lesions with the underlying condition of hyperglycemia. The oral lesions were treated using the local application of clotrimazole gel and the discomfort subsided with Aceclofenac. The underlying condition was treated by a general physician who prescribed the tablet metformin 500mg. The patient was educated about the predisposing condition, and motivated to make some lifestyle changes and to maintain a proper diet.

## Introduction

Diabetes mellitus (DM) is a common endocrine condition that damages the immune system, making the patient more susceptible to infections. Several mechanisms are at play, including a decrease in T-lymphocyte numbers, a reduction of neutrophil activity, an increase in leukocytes that have undergone programmed cell death, and a decrease in cytokine production [1]. In diabetic individuals, the hyperglycemic environment exacerbates all of these issues. Recurrent candidal infections are a well-known indicator of DM and can occasionally help determine a person's pre-diabetic status [2-5]. When a person's fasting plasma glucose falls between 100-125 milligrams/decilitre, it is hypothesized that they have impaired glucose tolerance or pre-diabetes [6].

Patients with diabetes mellitus are more susceptible to opportunistic infections and multiple organ damage, which impairs their resistance to invasive pathogens like Candida species that have a wide geographic distribution pattern [4]. In addition to diabetes, a number of additional aspects, including wearing dentures, smoking, experiencing xerostomia, using steroids, and using broad-spectrum antibiotics, might make a person more susceptible to oral candidal colonization. If dental hygiene is neglected, oral candidiasis can develop quickly. Patients with pre-existing illnesses such as diabetes or HIV are more susceptible to this condition [5]. Although oral candidiasis can affect anybody, it is more common in elderly individuals and newborns due to their weakened immune systems, as well as in those with specific ailments, suppressed immune systems, and those on certain drugs. Candida species isolates have much greater haemolytic and esterase enzymatic activity due to higher blood glucose concentrations; this may be a factor in diabetes individuals' elevated enzyme activity. The present article presented a case of a 52-year-old female patient who reported recurrent multiple plaque-like lesions with the underlying condition of hyperglycemia.

## Case presentation

A 52-year-old female patient presented to the oral medicine and radiology outpatient department with the complaint of multiple painful ulcers in the oral cavity since 15 days which were initially smaller in size and less severe and have gradually progressed to the current size and are more severe. The patient gave a history of recurrent ulcerations for two months. The patient was in extreme discomfort and was unable to eat or drink due to the ulcerations. The patient was even unable to speak properly and gave a history of increased salivation. The patient took some medications on her own for four days but did not get any relief. The patient did not report any relevant systemic disorders. The patient had no deleterious habit. The overall general examination of the patient did not detect any abnormality and was well-oriented to time, place, and person. On extraoral examination, the patient's face was grossly symmetrical. On lymph node examination, both right and left submandibular lymph nodes were palpable of size 1.5 x 1.5 centimeters approximately which were firm, mobile, and tender.

**Figure 1:**
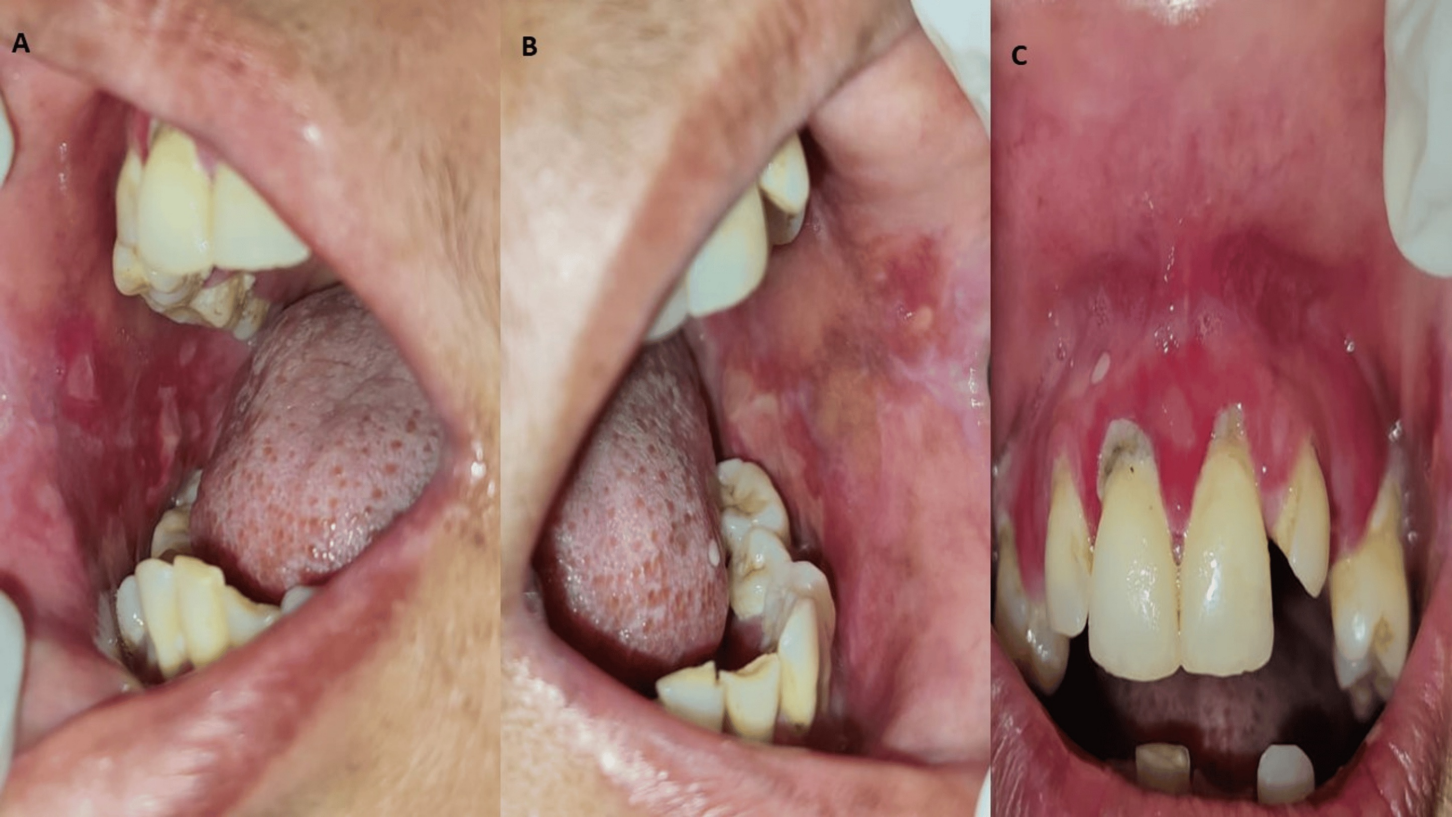
Multiple plaque-like lesions of varying size with erythematous mucosa A: shows the right buccal mucosa depicting multiple plaque-like lesions of varying size with erythematous mucosa B: shows the left buccal mucosa depicting multiple plaque-like lesions of varying size with erythematous mucosa C: shows the maxillary anterior region depicting inflamed marginal gingiva and diffused erythema

On intraoral examination, diffused, multiple plaque-like lesions were seen on the right and left buccal mucosa with diffused erythema. The size of the lesions varies from 0.5 x 0.5 centimeters to 2 x 2 centimeters approximately. The margins of the lesion were slightly elevated and diffused. The surface was covered with slough and the periphery was erythematous. On palpation, tenderness was present and consistency was firm with no induration on the edges. The marginal gingiva was inflamed with diffuse erythema (Figure 1).

**Figure 2:**
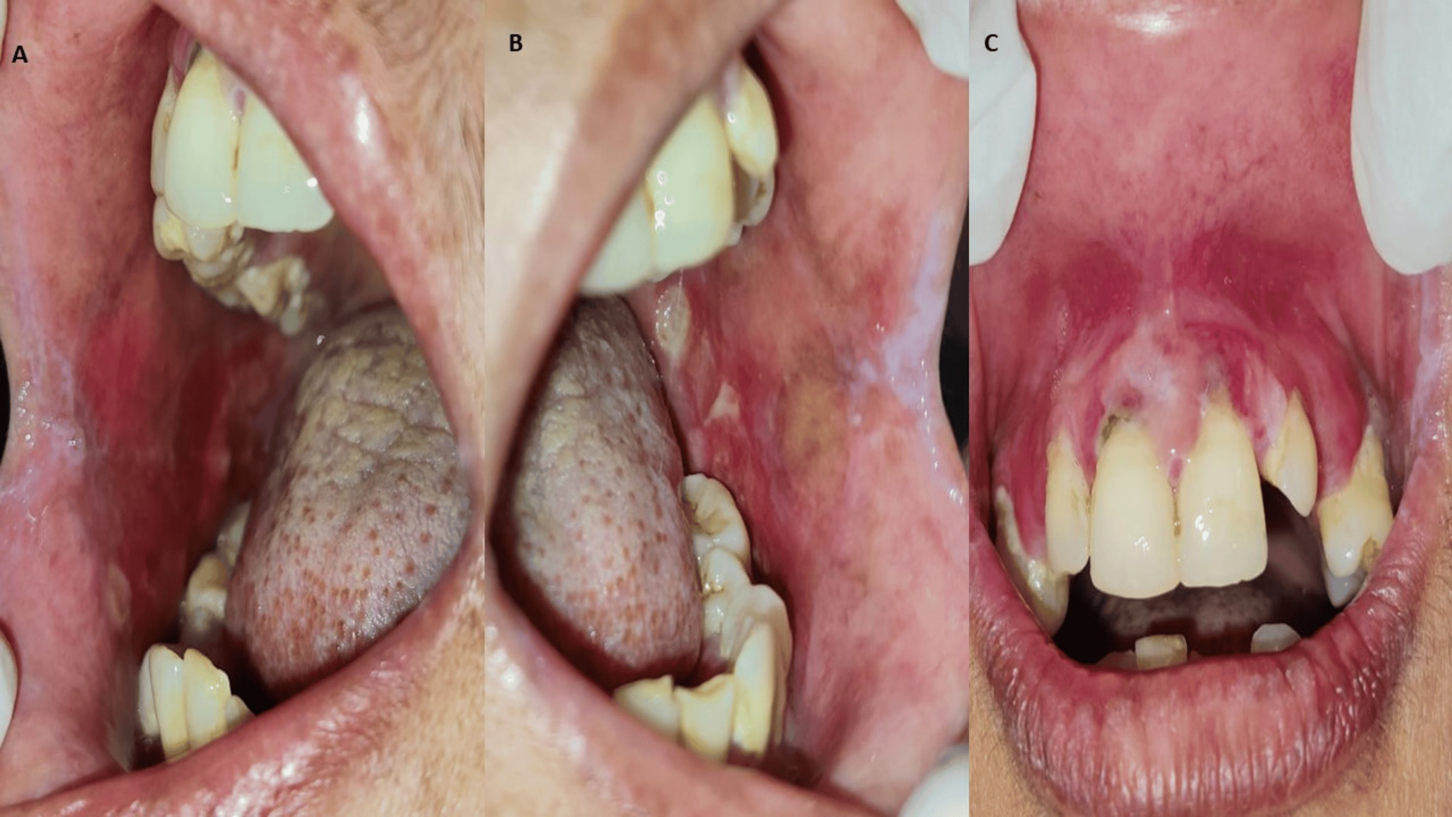
Reduction in the size and severity of the oral lesion and overall reduction in the erythema of oral mucosa seven days after first visit A: shows the right buccal mucosa depicting a reduction in the size and severity of the oral lesion and overall reduction in the erythema of oral mucosa B: shows the left buccal mucosa depicting a reduction in the size and severity of the oral lesion and overall reduction in the erythema of oral mucosa C: shows the maxillary anterior region depicting an overall reduction in the erythema of oral mucosa

**Figure 3:**
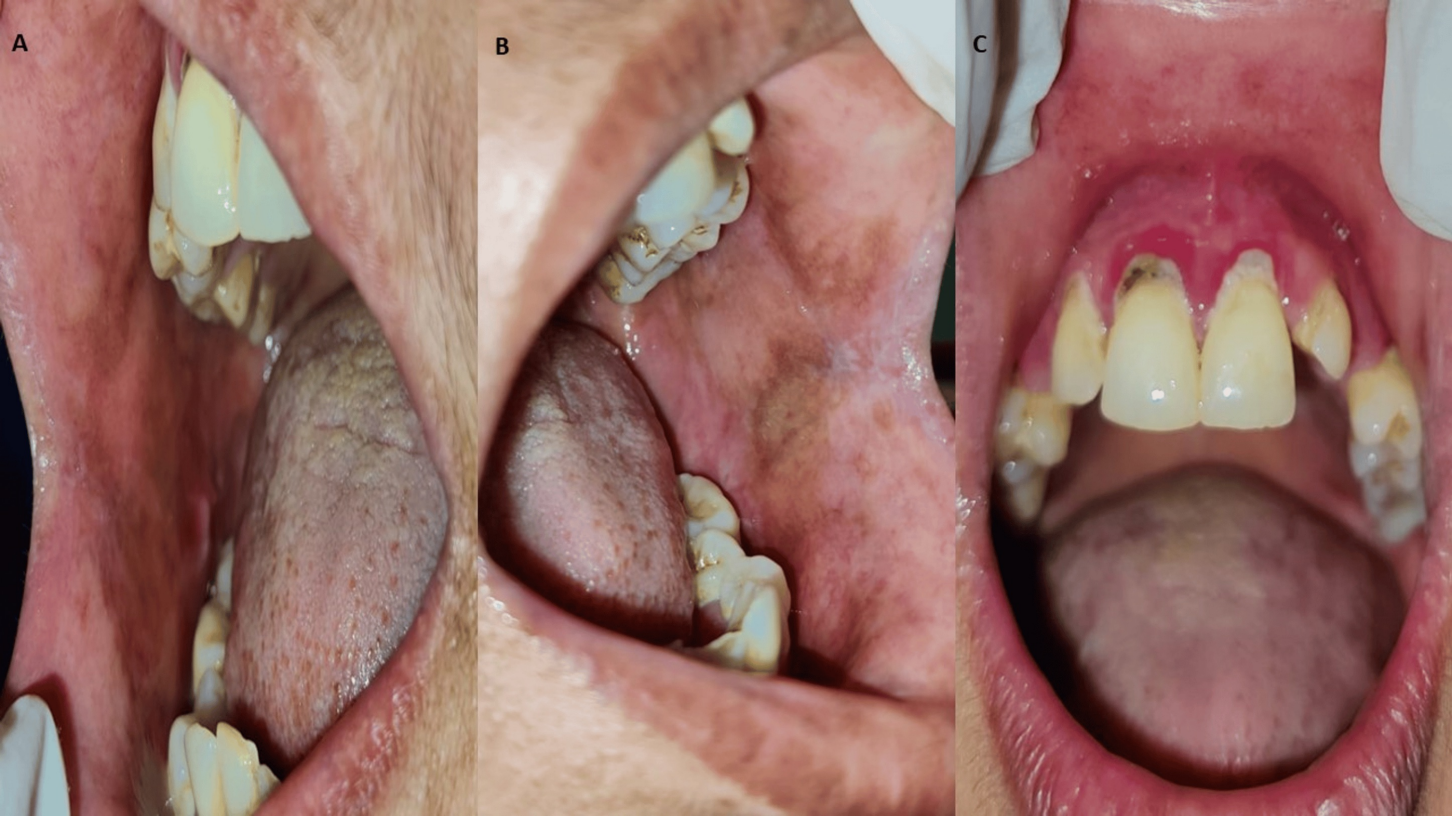
Complete healing of the plaque-like lesions in the oral cavity A: shows the right buccal mucosa depicting complete healing of the plaque-like lesions B: shows the left buccal mucosa depicting complete healing of the plaque-like lesions C: shows the maxillary anterior region depicting complete recovery of lesions.

Other findings include missing 23, 36, and grade II mobility with 16, 47. There was generalized attrition of teeth and generalized gingival recession. The overall oral hygiene of the patient was poor with the presence of calculus. Considering the clinical presentation of the patient, the provisional diagnosis of the patient was chronic plaque-like oral candidiasis. To relieve the patient’s oral symptoms the patient was prescribed tablet Fibronac (dispersible Aceclofenac) before meals, tablet Fibrowin (myoinositol) twice daily and local application of Candid mouth paint (clotrimazole) thrice a day for seven days. The patient was also advised to maintain oral hygiene. Aceclofenac reduces the pain and inflammation in the oral lesions, clotrimazole reduces the microbial load in the oral cavity whereas myoinositol lowers the risk of metabolic disorders. The patient was referred to a physician and advised for blood sugar investigations which revealed fasting blood sugar raised to 150.3 milligrams/decilitre and post-meal blood sugar to be 191.4 milligrams/decilitre. After knowing the underlying cause of the patient’s immunosuppressed condition, the final diagnosis was chronic plaque-like oral candidiasis due to uncontrolled type II diabetes mellitus. The patient was prescribed with tablet metformin 500 mg once a day. The patient was educated about the predisposing condition, and motivated to make some lifestyle changes and to maintain a proper diet. The patient was recalled for her second visit after seven days to review the oral lesion which revealed a reduction in the size and severity of the lesion. The overall erythema of the oral mucous membrane was reduced (Figure 2). The patient was recalled after 15 days for the review of the oral lesions. All the lesions of the oral cavity were resolved and healed (Figure 3).

The patient was again advised to investigate her blood sugar which revealed the fasting blood sugar to be 118.5mg/dl and post-meal blood sugar was 146.4mg/dl. The patient was advised to continue tablet metformin 500 mg once daily and to maintain her diet and lifestyle changes.

## Discussion

DM is a noncommunicable disease (NCD) and one of the most common endocrine disorders worldwide, according to the International Diabetes Federation. In Southeast Asia, it is also the most prevalent NCD [7]. Uncontrolled diabetes makes a person more susceptible to both systemic and superficial infections, and oral candidal infections are a prevalent ailment in individuals with this condition [4,8]. A case-control research conducted in 2019 by Sampath et al. found that co-carriage of various yeast species is a common finding in the study population and that the oral infestation of Candida in the Sri Lankan cohort of diabetics is much greater than in their healthy counterparts [3].

The fungus Candida, which resembles yeast, is the cause of oral candidiasis. Redness in the mouth and throat, a painful, burning sensation in the mouth, an unpleasant taste or loss of taste, and white patches (plaques) in the mouth that are often wipeable, leaving behind red areas that may bleed slightly are all signs of oral thrush [9]. Oral thrush seldom causes problems in people with good immune systems, but in severe cases, it can migrate to the oesophagus. The most prevalent species of Candida albicans, as well as those of C. tropicalis, C. glabrata, C. guillierimondii, C. lusitaniae, C. krusei, C. parapsilosis, C. stellatoidea, and C. pseudotropicalis, are important species implicated in oral candidiasis [7,10].

Candida species may colonize mucosal surfaces, including those of the oral cavity, gastrointestinal system, respiratory tract, and genitourinary tract. They constitute a normal human microbiota [11]. On the other hand, Candida species have the ability to coexist as a commensal or to turn asymptomatic colonies into infectious ones. Since the fungus and the host's present immunological state are intricately linked, the pathogenic potential of Candida species and their colonization factors depend on host-related immune variables. This interaction is the primary determinant of either commensalism or parasitism. Numerous established variables influence homeostasis and increase the susceptibility to Candida colonization. These elements facilitate its change from commensal to virulent. Several causes contribute to this phenomenon, such as the sticky nature of Candida species on the epithelial cell surfaces of the host, increased salivary glucose levels, decreased salivary flow rates, deterioration of the microvasculature, and a compromised neutrophil immunological response to candidacidal activities [12]. Oral thrush, periodontitis, and dental caries have also been linked to high oral candidal colonization in type II diabetes.

The clinical symptoms and history are the primary basis for the diagnosis; further testing is only necessary in complex instances. The clinical manifestations of oral mucosal candidiasis are diverse. Treatment of a candidal infection requires the identification of any local or systemic causal causes and their potential resolution. For topical use, the preferred medications are miconazole and nystatin [13-15]. Triazoles are effective systemic medicines; examples are fluconazole and itraconazole. The type of candidiasis and the existence of predisposing variables have a major impact on the dosage and length of therapy. Identifying oral candida lesions and developing efficient treatment plans are the primary responsibilities of any dental professional [9]. A complete medical history must be documented to diagnose this clinical problem. Predisposing variables must be addressed or eliminated wherever possible [14,15].

In 2020, Nurmansyah et al. reported a link between hyperglycemia and Candida albicans infection, which results in oral candidiasis in individuals with diabetes mellitus [8]. Antifungal agents should be applied locally in the form of suspension or pomade, mycological cultures should be evaluated to support clinical diagnosis and etiological factors that may cause the disease should be researched. Keser et al. published a case series of oral candidiasis in 2021 [7]. To enable early detection of infectious illnesses and ensure the timely beginning of therapy, the dentist must have a thorough understanding of the oral symptoms of diabetes. Given that the mouth is the body's entryway and that poor oral hygiene can cause several systemic disorders, it is essential to inform and encourage people to practice proper oral hygiene.

## Conclusions

The most prevalent metabolic syndrome condition resulting from lifestyle choices in current society is DM. Individuals diagnosed with DM are more susceptible to Candida albicans colonizing their urinary system and/or vaginal mucosa. The increased susceptibility of diabetics to acquire candidiasis relative to the healthy population has prompted much research on the relationship between DM and candidiasis. In the present case report, the patient presented with oral manifestations of candidiasis which was later diagnosed with uncontrolled type II diabetes mellitus. The oral manifestations of the patient were treated with antifungal drugs and analgesics whereas the underlying condition of DM was controlled by lifestyle changes and maintenance of diet.
